# Clinical features and treatment outcomes of acute-onset endophthalmitis caused by *Staphylococcus lugdunensis*: a case series

**DOI:** 10.1186/s12348-026-00568-z

**Published:** 2026-02-07

**Authors:** Justin A. Chen, Michael Y. Zhao, Lauren C. Kiryakoza, Salomon Merikansky, Landon J. Rohowetz, Darlene Miller, Harry W. Flynn Jr.

**Affiliations:** Department of Ophthalmology, Bascom Palmer Eye Institute, University of Miami Health System, 900 NW 17th St, Miami, FL 33136 USA

**Keywords:** Acute-onset endophthalmitis, *Staphylococcus lugdunensis*

## Abstract

**Purpose:**

To report a series of patients with acute-onset endophthalmitis caused by culture-proven *Staphylococcus lugdunensis* and to provide an update on the microbiologic susceptibility and clinical outcomes resulting from this organism.

**Observations:**

This study included 6 eyes of 6 patients. The etiologies included cataract surgery (4), intravitreal injection (1), and posterior segment surgery (1). All isolates (100%) of *S. lugdunensis* demonstrated sensitivity to vancomycin with minimal inhibitory concentration (MIC) values ranging from ≤ 0.5 to 1 mcg/mL and to moxifloxacin with MIC values ranging from ≤ 0.25 to 1 mcg/mL. One of 6 isolates demonstrated intermediate resistance to gentamicin with a MIC value of 8 mcg/mL. Four of 6 patients underwent pars plana vitrectomy following initial treatment with intravitreal antibiotics, and 1 eye underwent vitrectomy as initial management. Best-corrected visual acuity at last follow-up examination was*≥* 20/150 in 5/6 (83.3%) of cases and *≥* 20/40 in 3/6 (50%) of cases.

**Conclusion and importance:**

In this study, patients with acute-onset endophthalmitis caused by *S. lugdunensis* had relatively good outcomes, similar to a previous case series. Vancomycin continues to have consistent coverage against *S. lugdunensis* and remains an empiric therapeutic option for acute-onset endophthalmitis.

## Introduction

Acute-onset exogenous endophthalmitis is a rare but vision-threatening infection that can occur following intraocular procedures. Coagulase-negative *Staphylococci* are the most common causative organisms, accounting for approximately 70% of cases of postprocedural endophthalmitis [1]. Of the group, the 2 most common isolates cultured are *Staphylococcus epidermis* (81.9%) and *Staphylococcus lugdunensis* (5.9%) [2]. *Staphylococcus lugdunensis* may have been previously under-reported, as advances in microbiological laboratory techniques have allowed for improved identification of less prevalent coagulase-negative *Staphylococci* species [3]. Investigations into acute-onset endophthalmitis caused by *S. lugdunensis* have shown a wide variability of visual outcomes following appropriate intervention. This case series aims to provide an update on the clinical presentation, antibiotic susceptibility patterns, and treatment outcomes of patients diagnosed with acute-onset endophthalmitis due to *S. lugdunensis*.

## Findings

A review of all patients with a diagnosis of acute-onset endophthalmitis at the University of Miami, Bascom Palmer Eye Institute Microbiology Department from January 2015 through April 2025 was performed. Patients were included in this study if vitreous and/or aqueous samples taken at the time of diagnosis identified isolates of *S. lugdunensis*. In total, 7 patients (6 men, 1 woman; mean age 64.9 years) were identified to have met study criteria. One immunocompromised patient with a history of primary intraocular lymphoma treated with systemic chemotherapy and radiation, complicated by radiation retinopathy and disseminated chorioretinal inflammation, presented with a 5-year history of gradual decline in visual acuity that later progressed from 20/400 to light perception over the course of 1 month. This patient subsequently underwent PPV due to concern for lymphoma recurrence, with vitreous cassette cultures identifying *S. lugdunensis*. Anterior chamber cultures obtained 4 days post-vitrectomy again confirmed growth of *S. lugdunensis*. This patient was removed from data analysis as he was unique compared to the other patients in how endophthalmitis was acquired.

**Fig. 1:**
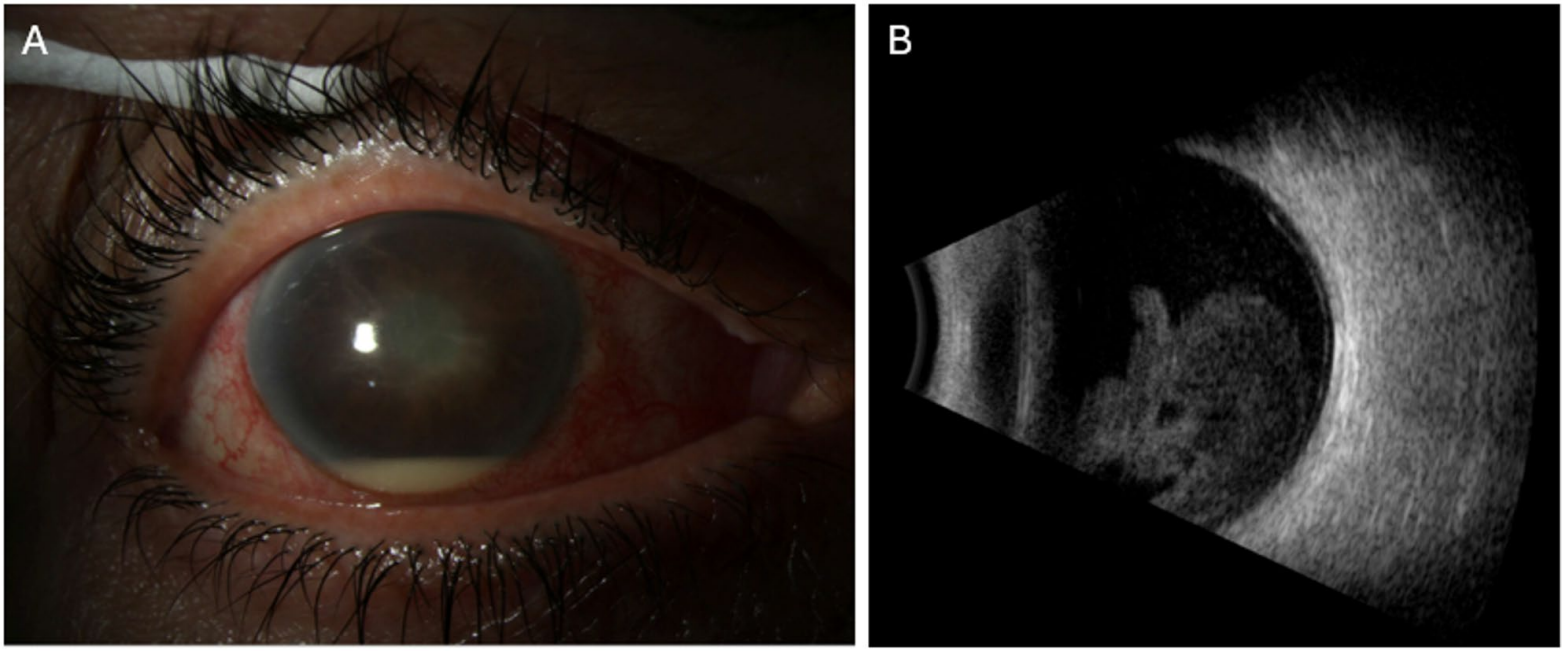
Patient 5: A 71-year-old female presented 4 days after cataract surgery in the right eye. Visual acuity was light perception. (**A**) Examination demonstrated diffuse conjunctival injection, corneal edema, and a hypopyon. (**B**) B-scan ultrasound revealed dense mobile vitreous opacities. The patient underwent vitreous tap and injection with vancomycin, ceftazidime and dexamethasone. Best-corrected visual acuity at last follow-up was 20/40.

**Fig. 2:**
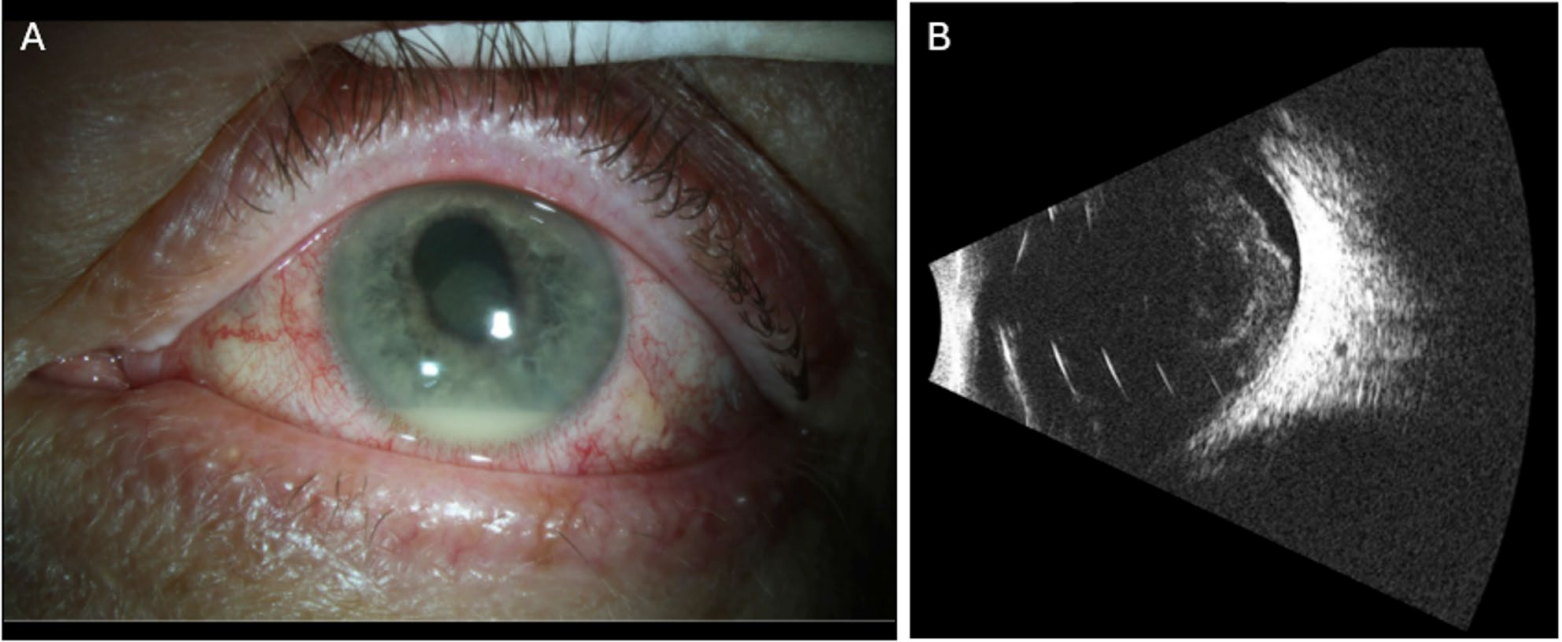
Patient 6: A 64-year-old male presented 5 days after receiving intravitreal bevacizumab in the left eye. Visual acuity was hand motions. (**A**) Examination demonstrated diffuse conjunctival injection, corneal infiltrate, and a 2 mm hypopyon. (**B**) B-scan ultrasound demonstrated dense vitreous debris and an incomplete posterior vitreous detachment. The patient underwent vitreous tap and injection with vancomycin, ceftazidime and dexamethasone and eventual pars plana vitrectomy with repeat injection of intravitreal vancomycin, ceftazidime and dexamethasone. Best-corrected visual acuity at last follow-up 6 days after initial presentation was hand motions.

The remaining 6 patients (5 men, 1 woman; mean age 62.4 years) all presented with acute-onset exogenous endophthalmitis with culture-proven *S. lugdunensis*. Four patients had undergone cataract extraction with intraocular lens implantation and presented on average 7.0 days after intraocular surgery. On examination, patients typically exhibited significant vision loss, conjunctival injection, hypopyon, fibrin in the anterior chamber, and marked vitreous opacities (Fig. 1). Patient 2 presented 8 days after cataract surgery, complaining of a tender eye but a visual acuity of 20/25. Examination was significant for diffuse conjunctival injection and significant cells in the anterior chamber. The patient later represented two days later with hand motion vision and new 1.5 mm hypopyon and dense vitreous debris on exam. Patient 6 presented 5 days after receiving an intravitreal injection of bevacizumab for proliferative diabetic retinopathy and diabetic macular edema (Fig. 2). Visual acuity was hand motions and exam was significant for diffuse conjunctival injection and hypopyon. B-scan ultrasound revealed dense, mobile vitreous opacities. Patient 3 presented with a visual acuity of light perception 3 days after pars plana vitrectomy (PPV) for visually significant vitreous opacities. Due to the abrupt and significant loss of vision following surgery, the patient underwent emergent PPV. The remaining patients were initially treated with vitreous aspiration and intravitreal antibiotics, most commonly vancomycin 1.0 mg and ceftazidime 2.25 mg. Patient presentation, clinical data and initial management are summarized in Table 1.

**Table 1:** Clinical presentations, interventions, and treatment outcomes of patients with acute-onset endophthalmitis caused by *S. lugdunensis*

Patient	Age/ Sex/Eye	History	Co-Morbidities	BCVA @ Presentation	Initial Treatments	Additional Treatments	BCVA @ Last Follow-up	Comments
1	50/M/OD	CE-IOL	None	HM @POD#11	Day 0: IV van, cef (POD#11)	YAG of fibrin and membrane @ POD#14 (Day 3)	20/150	LTFU POD#17 (Day 6)
2	53/M/OS	CE-IOL	Vitreous cassette from PPV grew MDR Serratia marcescens	20/25 @POD#8	Day 0: IV van, cef, vori (POD#8) Day 3: PPV/FAX/1000cs SO, IV vanc, cef (POD#11)	PPV/SOR/FAX @ POM#3	20/70	BCVA HM @POD#10 (Day 2) NVS ERM noted POM#4
3	70/M/OD	PPV for visually significant PVD	Allergic to amoxicillin	LP @POD#3	Day 0: PPV/1000cs SO, IV van, ami, dex (POD#3)	PPV/SOR/AC washout @ POW#6	20/30	Recurrent CME noted POM#6
4	64/M/OS	CE-IOL	None	LP @POD#5	Day 0: IV van, cef, dex (POD#5) Day 1: PPV with IV van, cef, dex (POD#6)	None	20/20	None
5	71/F/OD	CE-IOL	POAG	LP @POD#4	Day 0: IV van, cef, dex (POD#4)	PPV for debulking vitreous opacities @ POM#1	20/40	NVS ERM noted POM#2 NVS temporal operculated hole noted POY#2
6	64/M/OS	IV bevacizumab for DME	PDR with DME receiving IV bevacizumab	HM @POD#5	Day 0: IV van, cef, dex (POD#5) Day 1: PPV with IV van, cef, dex (POD#6)	None	HM	LTFU POD#11 (Day 6)

Abbreviations: AC, anterior chamber; Ami, amikacin; BCVA, best corrected visual acuity; Cef, ceftazidime; CE, cataract extraction; CME, cystoid macular edema, Cs, centistoke; Dex, dexamethasone; DME, diabetic macular edema; ERM, epiretinal membrane; FAX, fluid-air exchange; HM, hand motion; IOL, intraocular lens; IV, intravitreal; LP, light perception; LTFU, lost to follow-up; MDR, multi-drug resistant; NVS, not visually significant; OD, right eye; OS, left eye; PDR, proliferative diabetic retinopathy; POAG, primary open angle glaucoma; POD, post-operative day; POM, post-operative month; POW, postoperative week; POY, postoperative year; PPV, pars plana vitrectomy; PVD, posterior vitreous detachment; SO, silicone oil tamponade; SOR, silicone oil removal; Van, vancomycin; Vori, voriconazole; YAG, yttrium aluminum garnet laser capsulotomy.

Of the 6 patients who presented, patient 1 and patient 6 were both lost to follow-up 6 days after initial evaluations; last documented best corrected visual acuity (BCVA) was 20/150 and hand motions, respectively. Of the remaining 4 patients, BCVA was favorable; 100% (4/4) had BCVA *≥* 20/70 and 75% (3/4) had BCVA ≥ 20/40 at last follow-up. Five of the 6 patients were initially treated with intravitreal antibiotics, while 1 patient underwent vitrectomy as initial management. Of the 5 patients who received intravitreal antibiotics, 4 patients subsequently underwent PPV. Subsequent treatments and clinical outcomes are described in Table 1.

*S. lugdunensis* was isolated in microbiologic cultures in all 6 cases, with antibiotic susceptibilities and minimum inhibitor concentrations (MIC) in mcg/mL documented in Table 2. All isolates were sensitive to vancomycin with MIC values ranging from ≤ 0.5 to 1 mcg/mL and to moxifloxacin with MIC values ranging from ≤ 0.25 to 1 mcg/mL. Five isolates were sensitive to gentamicin with MIC values of ≤ 0.5 mcg mL, with 1 demonstrating intermediate resistance with an MIC of 8 mcg/mL Of the 6 total isolates, 5 were tested for oxacillin susceptibilities, with 2 demonstrating resistance with MIC values ≥ 4 mcg/mL.

**Table 2:** Antibiotic susceptibilities and minimum inhibitory concentrations (mcg/ml) of patients with acute-onset endophthalmitis caused by *S. lugdunensis*

Patient #	Vancomycin	Gentamicin	Oxacillin	Moxifloxacin		Clindamycin		
1	S (≤ 0.5)	S (≤ 0.5)	S (2)	S (≤ 0.25)		S (0.25)		
2	S (≤ 0.5)	S (≤ 0.5)	N/A	S (≤ 0.25)		S (≤ 0.12)		
3	S (1)	I (8)	R (≥ 4)	S (≤ 0.25)		S (0.25)		
4	S (≤ 0.5)	S (≤ 0.5)	S (2)	S (≤ 0.25)		S (0.25)		
5	S (0.5)	S (0.5)	S (2)	S (1)		S (0.25)		
6	S (1)	S (≤ 0.5)	R (≥ 4)	S (≤ 0.25)		S (0.25)		

Abbreviations: I, Intermediate; R, Resistant; S, Sensitive. Minimum inhibitory concentration, if available, is in parentheses in mcg/ml.

## Discussion

*Staphylococcus lugdunensis* is known to cause an array of clinical infections including skin and soft tissue infections but can also demonstrate marked pathogenicity in cases of osteomyelitis, prosthetic joint infections, and infective endocarditis [3]. The virulence and aggressive clinical course of non-ocular cases of *S. lugdunensis* is well documented and has been compared to that of *Staphylococcus aureus* [4]. Studies evaluating non-ocular infections of *S. lugdunensis* have demonstrated greater pathogenicity than other coagulase-negative *Staphylococci* species, often requiring more intensive treatment [4]. 

There has been a wide range in reported visual outcomes in patients with acute-onset exogenous endophthalmitis caused by *S. lugdunensis*. In a retrospective study, Murad-Kejbou et al. documented 3 patients with limited visual recovery, although these poor outcomes may have had limited visual potential due significant co-morbidities (e.g., end-stage glaucoma and advanced age-related macular degeneration) [5]. Another case series by Chiquet et al. described 5 cases, noting poor visual outcomes after PPV potentially due to post-operative retinal detachment [6]. However, a retrospective case series by Chen et al. reported 2 cases of acute-onset exogenous endophthalmitis caused by culture-proven *S. lugdunensis* from Taiwan; both patients achieved a BCVA ≥ 20/50 following intravitreal antibiotics and subsequent PPV [7]. 

In this review, visual outcomes were encouraging in patients with acute-onset endophthalmitis caused by *S. lugdunensis*, especially in those who were able to attend longitudinal follow-up. The poor visual acuity observed in 2 patients may reflect the brief duration of follow-up, which did not extend beyond 1 week. These favorable outcomes are similar to the previous retrospective case series of 6 patients by Garoon et al. At last follow-up, Garoon et al. reported 5 of 6 patients (83.3%) with BCVA ≥ 20/100 and 3 (50%) with BCVA ≥ 20/40 [8]. It was noted that of the 3 patients who had more limited visual recovery, 1 patient experienced corneal decompensation requiring keratoplasty and intraocular lens repositioning, and 2 other patients had open globe injury in addition to endophthalmitis, followed by macula off retinal detachment, aphakia, and irregular astigmatism [8]. However, interpretation is ultimately limited by the small cohort size and incomplete follow-up.

There are multiple factors that may affect the visual outcomes of patients with endophthalmitis. Visual outcomes tend to be poorer if the identified organism is more virulent and/or has a higher rate of antibiotic resistance (e.g., *Enterococci*) [9]. However, prompt diagnosis of suspected endophthalmitis and appropriate treatment can be vision saving. Initial treatment tends to be either vitreous tap and injection of antibiotics or PPV with intravitreal antibiotics [10]. An investigation on antibiotic-resistant endophthalmitis by Choi et al. demonstrated that patients who underwent early vitrectomy (< 24 h of symptom onset) following initial intravitreal antibiotics achieved better final visual acuity at the last follow-up visit than those who received delayed vitrectomy [11]. Close monitoring is important to monitor clinical progression and determine the need for repeat intravitreal antibiotics or vitrectomy.

Data on the microbiologic susceptibility of *S. lugdunensis* isolated from intraocular sources remain relatively limited. All isolates in this review demonstrated sensitivity to vancomycin and moxifloxacin, which is consistent with the sensitivities reported by Garoon et al.^4^ Differing from Garoon et al., 1 isolate in this review demonstrated intermediate resistance to gentamicin. A large epidemiologic study from United States tertiary care centers by Palumbo et al. investigating *S. lugdunensis* isolated from various non-ocular sources demonstrated comparable susceptibility patterns. However, the authors reported that all isolates demonstrated sensitivity to gentamicin [5]. To our knowledge, this represents the first documented instance of gentamicin resistance across all isolates of S. lugdunensis reported in the USA.

Surveillance for aminoglycoside resistance in endophthalmitis caused by *S. lugdunensis* may be warranted, and use of intravitreal amikacin in endophthalmitis should be guided by susceptibility testing. As reflected in this review, *S. lugdunensis* remains susceptible to vancomycin and fluoroquinolones with no documented resistance; these isolates demonstrated less overall antibiotic resistance relative to general susceptibility patterns [3, 5]. Despite concerns for rising antibiotic resistance with other *Staphylococcus* species, vancomycin continues to be effective against *S. lugdunensis* and should remain as a first-line antibiotic for treatment of acute-onset endophthalmitis caused by coagulase-negative *Staphylococci*.

## Data Availability

No datasets were generated or analysed during the current study.
